# Lethargy and quadraparesis as initial manifestations of covid-19 child: Case report

**DOI:** 10.1016/j.amsu.2022.104911

**Published:** 2022-11-17

**Authors:** Ali Hammed, Maysaa Badour, Sameer Baqla

**Affiliations:** a Tishreen University Hospital, Department of Neurosurgery. Lattakia, Syria; b Pediatric University Hospital, Damascus, Syria

**Keywords:** Lethargy, Quadraparesis, SARS-CoV-2

## Abstract

**Introduction:**

and importance: SARS-CoV-2 infection classically presents with fever and respiratory illness. However, neurological manifestations are also being reported in the literature. Transverse myelitis is caused by inflammation of spinal cord. There are various possible etiologies for this neurologic condition that include viral or bacterial infections.

**Case presentation:**

We present a case of 2 –year-old female complained of weakness of all four limbs and lethargy.She was febrile(39), respiratory rate 30/min and oxygensaturation of 89% on room air. Neurological examination revealed intact cranial nerves, GCS of 14/15 and upper and lower limbs weakness with medical research council(MRC) score of 2/5 Sensory examination showed decreased sensation of all modalities in lower limbs with a sensory level at T4.

**Clinical discussion:**

Laboratory results and cerebrospinal fluid (CSF) analysis showed normal values. Brain MRI was normal. An urgent Gadolinium-enhanced magnetic resonance imaging of the whole spine was done and revealed extensive diffuse hyper intense signal involving predominantly the grey matter of the upper cervical spinal cord. Mild enlargement and swelling of the cervical cord were also note. She was given pulse doses of IV methylprednisolone 30mg/kg followed by oral prednisolone for 40 days. She was also given IV gamma globulin 400mg/kg for five days. A marked improvement of his neurological deficit was noted over a period of 16 days after treatment.

**Conclusion:**

when a patient with myelopathy is systemically ill with fever andloss of consciousness, prompt investigation of the causative agent is needed for appropriate management. Even after the pandemic Status; COVID-19 should be considered a differential diagnosis in patients presenting with loss of consciousness, ataxia, convulsions, motor deficits, encephalitis, myelitis, or neuritis.

## Highlights

• acute myelitis as a neurological complication of COVID-19 infection
• SARS-CoV-2 infection classically presents with fever and respiratory illness.
• When transverse myelitis is a result of viral or bacterial infection, it is usually considered as an immune mediated response.

## 1 Introduction

The novel 2019 coronavirus epidemic first started in Wuhan, China and spread to almost all countries around the world, most of them currently struggling with the consequences of the disease. [1].

SARS-CoV-2 infection classically presents with fever and respiratory illness. However, neurological manifestations are also being reported in the literature. The neurological features of this infection include headache, dizziness, anosmia, taste disturbances, cerebrovascular accident, Guillain-Barre syndrome, acute encephalitis and acute transverse myelitis (ATM) [2,3].

Transverse myelitis is caused by inflammation of spinal cord. There are various possible etiologies for this neurologic condition that include viral or bacterial infections, multiple sclerosis, neuromyelitis optica, systemic autoimmune diseases and infarction. [4] When transverse myelitis is a result of viral or bacterial infection, it is usually considered as an immune mediated response [20].

Several cases of acute transverse myelitis are reported in association with this disease among the world. [[5], [6], 7, [8], [9]] [18].

We presented a case of acute myelitis as a neurological complication of COVID-19 infection that was admitted with quadraparesis and urinary retention.

This work has been reported in line with the SCARE criteria [19].

## 2 Case presentation

A previously healthy 2 –year-old female was admitted to the emergency department at pediatric hospital complaining of weakness of all four limbs and lethargy.

On admission, she was febrile(39), pulse rate 140/min, her blood pressure 9/6, respiratory rate 30/min and oxygensaturation of 89% on room air. Initial neurological examination revealed intact cranial nerves, GCS of 14/15 and upper and lower limbs weakness with medical research council(MRC) score of 2/5 in proximal and distal. She had absent deep tendon reflexes with bilateral planter extensor.

Sensory examination showed decreased sensation of all modalities in lower limbs with a sensory level at T4.

Patient complained of urinary retention and A Foleys catheter was inserted. almost 1.6 L of urine was drained.

Past medical and surgical history were unremarkable. Her parents were COVID- 19 positive. A chest x-ray in the frontal view was normal.

Laboratory results showed normal white blood cell count (8.2 ^3/UL). Hemoglobin was decreased (10 g/dl). Inflammatory markers C-reactive protein and the erythrocyte sedimentation rate were normal.

D-Dimer and coagulation profile were normal. Anticoagulant proteins; Protein C, Antithrombin III, and activated Protein C resistance were within normal limits.

Cerebrospinal fluid (CSF) analysis, with unremarkable findings (white blood cell count 0, red blood cell count 250, glucose 73, protein 37.4) and CSF culture was negative.

A PCR nasal swab gave a positive result for the novel COVID-19). Other viral PCR screening including Adenovirus, Epstein Barr virus, Herpes Simplex virus (type 1&2), Cytomegalovirus, and Human Immunodeficiency virus showed negative results. Serology of other viruses such as Influenza Virus A and B, Parainfluenza 1–4, Respiratory Syncytial virus, Enterovirus and Rhinovirus were also negative. Bacteria associated with acute myelitis such as: Chlamydia Pneumoniae, Bordetella Pertussis, Mycoplasma Pneumoniae, and Borrelia antibodies gave negative results.

**Fig. 1:**
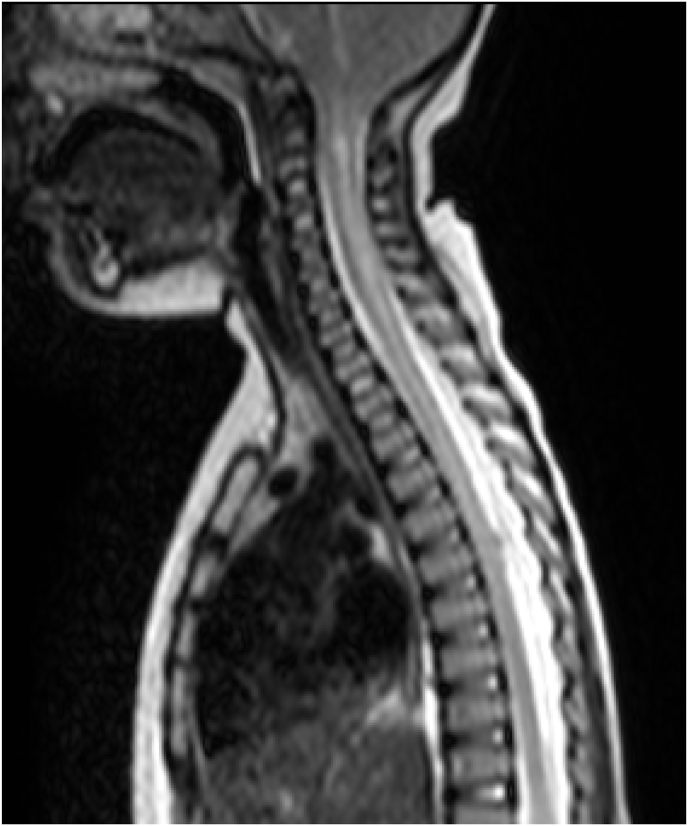
Gadolinium-enhanced magnetic resonance imaging of the whole spine was done and revealed extensive diffuse hyper intense signal involving predominantly the grey matter of the upper cervical spinal cord. Mild enlargement and swelling of the cervical cord were also noted.

Brain MRI was normal. Gadolinium-enhanced magnetic resonance imaging of the whole spine was done and revealed extensive diffuse hyper intense signal involving predominantly the grey matter of the upper cervical spinal cord. Mild enlargement and swelling of the cervical cord were also noted. (Fig. 1). Findings were suggestive of acute myelitis likely as direct damage or a sequalae of a post infectious process of the novel corona virus (COVID-19) as all other commonly associated viruses and immunological disorders were excluded.

After obtaining the patient's informed consent, treatment was planned by a consultant neurologist.

She was given pulse doses of IV methylprednisolone 30mg/kg followed by oral prednisolone for 40 days.

She was also given IV gamma globulin 400mg/kg for five days.

**Fig. 2:**
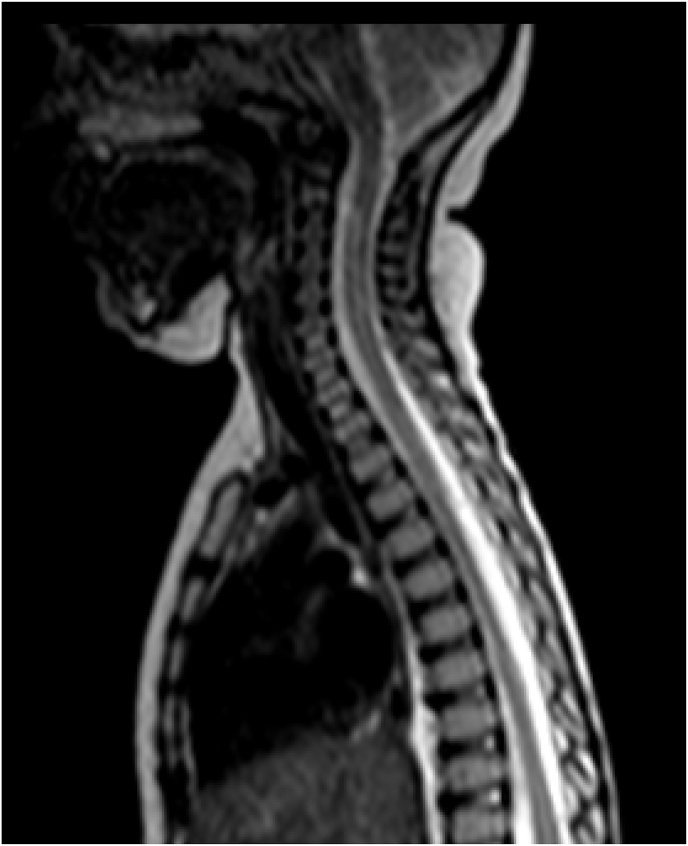
MRI T2 Sagittal showed partial resolution of the changes seen previously.

MRI whole spine was repeated on day 10, which showed partial resolution of the changes seen previously. Fig. 2.

A marked improvement of her neurological symptoms was noted after 16 days of treatment, she regained power inhis upper limb 4/5 and bilateral lower limbs 3/5.

## 3 Discussion

ATM is a focal inflammatory disorder of the spinal cord, characterized clinically by acute or subacute motor, sensory, and autonomic dysfunction [10]. Preceding nonspecific fever, nausea, or muscle pain, possibly indicating viral infection, are common [11]. ATM is associated with multiple sclerosis, systemic mixed connective tissue disorder, direct infection of the spinal cord (i.e., Mycoplasma or herpes simplex virus), and vascular causes (i.e., infarct or vascular malformation), but can also appear without an established etiology [12].

Given the recent and rapidly progressing nature of the pandemic, exact statistics are unknown, although estimates suggest that 80% of patients experience mild illness. Less appreciated still are the complications, sequelae, and long-term effects of COVID-19 [13].

The primary target of SARS-CoV-2 virus is respiratory epithelium via the angiotensin-converting enzyme-2 (ACE 2) receptor. ACE 2 receptors are also present in glial cells of brain and spinal neurons, and this could be a probable mechanism for dissemination of SARS-CoV-2 into the central nervous system [14,21].

Hypoxic damage and metabolic abnormalities, direct invasion by the virus or an exaggerated immune response tothe virus are proposed to cause neurological dysfunction [15]. Immune-mediated damage mainly occurs due to activation of inflammatory cells such as T lymphocytes and macrophages and subsequent overproduction of inflammatory cytokines, resulting in ‘cytokine storm’ [16]. Interleukin 6 (IL-6) causes further damage by causing endothelial dysfunction, activation of coagulation andcomplement cascade and ultimately results in organ dysfunction. The exact pathogenesis of ATM remains obscure and an exaggerated inflammatory response (‘cytokine storm’) related to a viral infection has been proposed as a possible cause.

The effect of IVIG could also relate to the presence of natural antibodies. IVIG also contains cytokines, and perhaps neutralizing antibodies; interestingly, antibodies against granulocyte macrophage colony-stimulating factor, interferon, interleukin 1, and interleukin 6 in immune globulin have biologic activity in vivo [17].

## 4 Conclusion

When a patient with myelopathy is systemically ill with fever andloss of consciousness, prompt investigation of the causative agent is needed for appropriate management.

Even after the pandemic Status; COVID-19 should be considered a differential diagnosis in patients presenting with loss of consciousness, encephalitis and other neurologic symptoms.

## Ethical approval

This study was not applicable for ethical approval.

## Sources of funding

This research did not receive any specific grant from funding agencies in the public, commercial, or not-for-profit sectors.

## Author contribution

Maysaa Badour: first author, data collection, writing the paper.

Ali Hammed (corresponding author): Writing the paper.

Sameer Baqla: Treatment and examination of the patient. Writing the paper.

## Registration of research studies

The case report at hand is not a first-in-man case report of a novel technology or surgical technique, therefore a registration of these case reports according to Declaration of Helsinki 2013 is not required.

## Guarantor

Ali Hammed.

## Consent for publication

Written informed consent was obtained from the patient for publication of this case report and accompanying images. A copy of the written consent is available for review by the Editor-in-Chief of this journal on request.

## Provenance and peer review

Not commissioned, externally peer-reviewed.

## Declaration of competing interest

All authors declared no conflict of interest.
